# Supplementary material for: Helicase-Like Transcription Factor HLTF and E3 Ubiquitin Ligase SHPRH Confer DNA Damage Tolerance through Direct Interactions with Proliferating Cell Nuclear Antigen (PCNA)
Source: Int J Mol Sci. 2020 Jan 21;21(3):693. doi: 10.3390/ijms21030693 (PMC7037221; doi:10.3390/ijms21030693)
Supplement: Supplementary file 1 [file ijms-21-00693-s001.pdf]

Supplementary material

S1

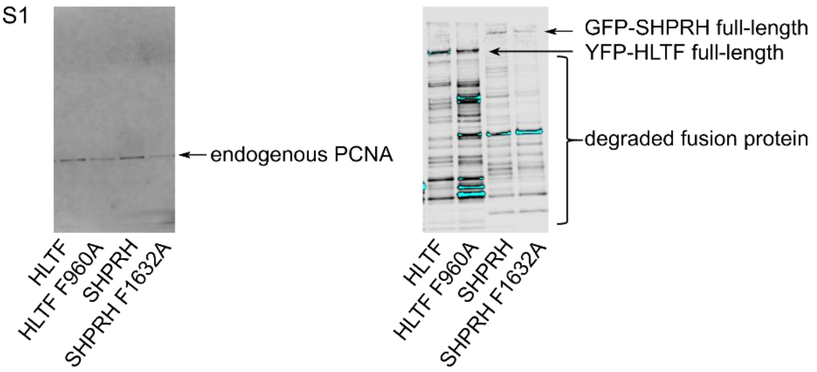

Figure S1. Mutation in APIM in HLTF or SHPRH reduced PCNA pulldown after DNA damage. Western blot analysis of proteins pulled down with α-GFP coupled beads from weakly crosslinked HEK293T cells after overexpression of YFP-HLTF, YFP-HLTF F960A, GFP-SHPRH, GFP-SHPRH F1632A and treatment with 50 μM MMS. Pulldown of endogenous PCNA (left panel, anti-PCNA), pulldown of the overexpressed proteins (right panel, anti-GFP).
